# Changes in and Patterns of Smoking Exposure in an Elderly Urban Population in Beijing: 2001–2010

**DOI:** 10.1371/journal.pone.0118500

**Published:** 2015-03-18

**Authors:** Shanshan Yang, Yao He, Miao Liu, Yiyan Wang, Lei Wu, Jianhua Wang, Di Zhang, Jing Zeng, Bin Jiang, Xiaoying Li

**Affiliations:** 1 Institute of Geriatrics, Chinese PLA General Hospital, 28 Fuxing Road, Beijing, 100853, China; 2 Beijing Key Laboratory of Aging and Geriatrics, Chinese PLA General Hospital, 28 Fuxing Road, Beijing, 100853, China; 3 State Key Laboratory of Kidney Disease, Chinese PLA General Hospital, 28 Fuxing Road, Beijing, 100853, China; 4 Department of Chinese Traditional Medicine and Acupuncture, Chinese PLA General Hospital, 28 Fuxing Road, Beijing, 100853, China; 5 Department of Geriatric Cardiology, Chinese PLA General Hospital, 28 Fuxing Road, Beijing, 100853, China; 6 Jinan Military Area CDC, Jinan, Shandong, 250014, China; School of Public Health of University of São Paulo, BRAZIL

## Abstract

**Objective:**

The study aims to explore the patterns and changes of active and passive smoking in the elderly population.

**Methods:**

Two cross-sectional surveys with representative samples of urban populations, aged between 60 and 95 years old, were conducted in 2001 and 2010 in Beijing. A current smoker was defined as a person who smoked a tobacco product at the time of the survey, and a passive smoker was defined as a person who had been exposed to smoke exhaled by a smoker for more than 15 minutes per day more than once per week.

**Results:**

A total of 2,277 participants in 2001 and 2,102 participants in 2010 completed the survey. The current smoking prevalence changed slightly in males (24.7 vs. 21.2%, P = 0.081), while the prevalence in females decreased significantly from 8.8% (95% CI: 7.3–10.3%) in 2001 to 4.1% (95% CI: 3.0–5.2%) in 2010 (P<0.001). The prevalence of passive smoking was 30.5% (95% CI: 28.6–32.4%) in 2001 and 30.0% (95% CI: 28.1–32.0%) in 2010. The main source of secondhand smoke switched from a spouse in 2001 to offspring in 2010. This trend was observed in both sexes. Passive smoking in males from a smoking spouse decreased from 5.7% to 2.4% (P<0.001), while that from smoking offspring increased from 7.3 to 14.5% (P<0.001). Passive smoking in females from a spouse decreased from 30.6 to 17.6%, while that from offspring increased from 5.3 to 15.4% (P<0.001).

**Conclusion:**

Offspring became the main source of secondhand smoke for the elderly. Our findings demonstrated the importance of implementing smoking prevention programs, to educate older adults who live with a smoking spouse and/or offspring.

## Introduction

Smoke exposure is one of the most serious health problems worldwide [1]. Smoking creates a heavy disease burden and is associated with a 50% higher all-cause mortality rate among men who are smokers [2]. Active smoking is currently the most preventable cause of death, disability and a variety of chronic diseases [3–9]. Furthermore, people who have never smoked can be exposed to the hazards of tobacco via passive smoking or secondhand smoking [10–15]. Passive smoking exposure costs billions of dollars in excess medical care every year [16].

There has been a trend toward a decrease in smoking prevalence in the United Kingdom[17] and the United States[18] due to the efforts of long-term tobacco control strategies; however, in France, although there was a long-term decrease in smoking prevalence prior to 2005, an increase was observed between 2005 and 2010 [17]. Further, in Nigeria, the smoking prevalence increased among adolescents [19]. Even in the United Kingdom, after a long-term decline, the overall smoking prevalence was still 24.9% [20].

China is the largest tobacco grower and consumer in the world [21], and the disease burden caused by tobacco smoking is serious [22, 23]. A study conducted in East Asia showed that the current smoking rate of Chinese adult men (20–69 years old) was 52.9% between 2008 and 2011[24]. Smoking does not only cause adverse health effects in adults, and its influence on health and quality of life might be greater in the elderly population[25,26]. Further, a previous study reported that, even in adults older than 60 years old, smoking cessation was still beneficial to health and improved life expectancy in elderly populations [27]. To find groups that have a higher risk of smoke exposure and to identify the best approach to support smoking cessation in the elderly, it is important to understand smoking exposure and patterns in elderly populations. However, there have been few data about smoking exposure status in elderly population; even in studies with large samples, the data on the elderly population have rarely been divided from those of other adults [20,24]. In China, data on the elderly have been rare as well. Wanshoulu district is a typical district of urban Beijing, and a study of the elderly population of this district could constitute a miniature of the Chinese elderly urban population and could provide primary relevant data.

Thus, this study compared the results of two surveys from the Beijing Wanshoulu district (a typical urban district of Beijing) in 2001 and 2010 to (1) describe the trends in the prevalence of smoking (active and passive) among the Wanshoulu district elderly population, as well as among social and demographic subgroups; (2) describe the characteristics of active and passive smokers; and (3) estimate the changes and patterns of active and passive smoke exposure in Beijing Wanshoulu elderly populations over a 10-year period.

## Design and Methods

### Study sample

**Fig 1:**
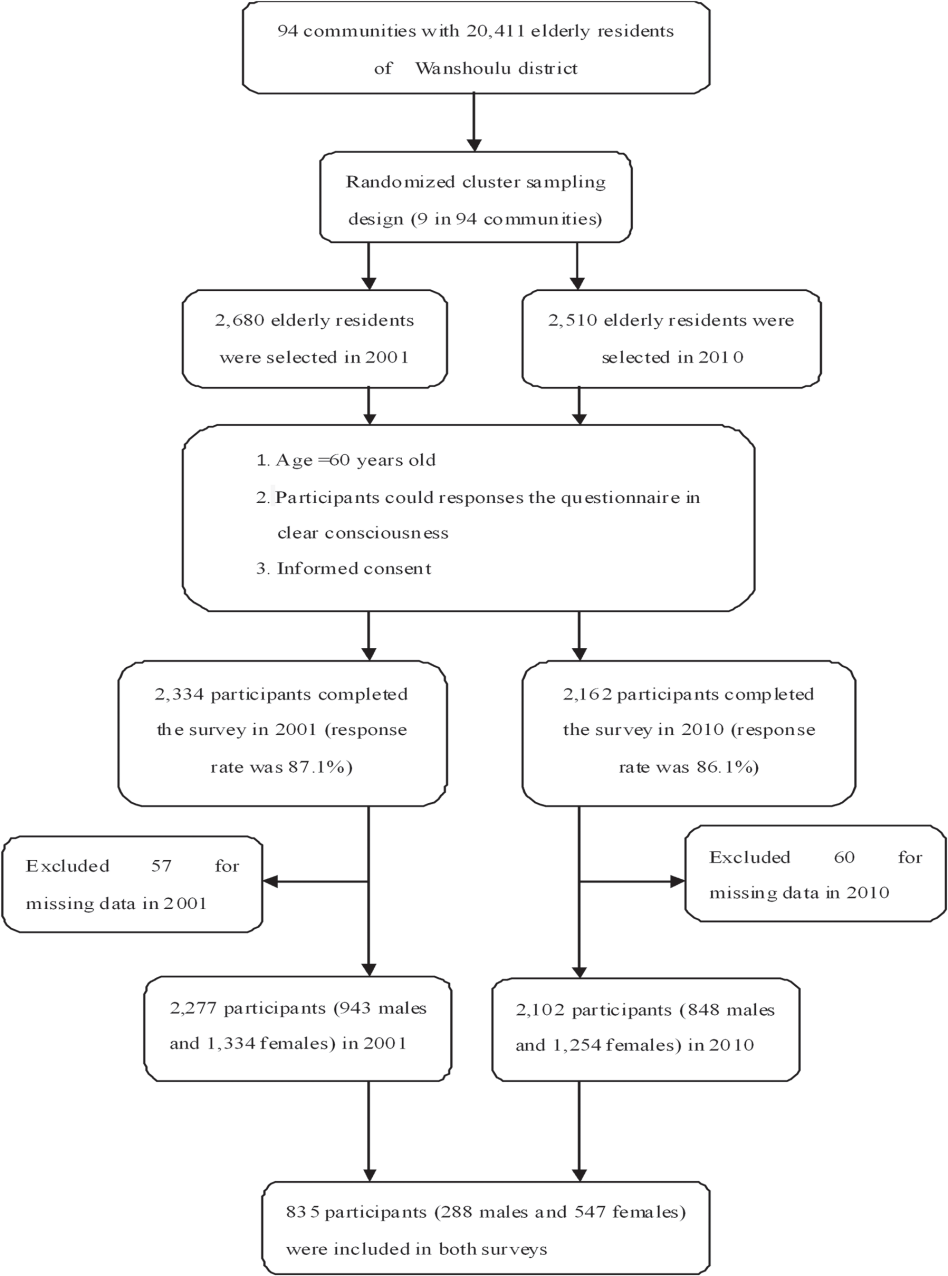
Flow diagram of the study population.

We conducted two population-based, cross-sectional surveys in 2001 and 2010, including an elderly population (≥60 years old) who lived in the Wanshoulu district as our participants. In 2001, as described in our previous study [28], there were 94 communities with 20,411 elderly urban residents (≥60 years old) in Wanshoulu district; we used the randomized cluster sampling method and randomly selected 9 of the 94 communities first, all of the households with elderly residents (n = 2,680) in the 9 communities were selected second, and from each household, we selected one elderly resident randomly as our participant. A total of 2,334 participants (943 males and 1,391 females) completed the survey (the response rate was 87.1%); after excluding 57 people with missing smoking exposure data, a total of 2,277 participants (943 males and 1,334 females) were included in our study as participants in 2001. For the 2010 survey, we used the same sampling structure and surveyed the same district. There were 2,510 participants selected in 2010, and 2,162 participants completed the survey (the response rate was 86.1%). After excluding 60 people with missing smoking exposure data, a total of 2,102 participants (848 males and 1,254 females) were included in our study as participants in 2010. We involved approximately 10% of the total number of elderly residents in the Wanshoulu district in our study in both surveys, and a total of 835 participants (39%) were included in both surveys (Fig. 1). Trained interviewers interviewed the participants face-to-face and completed a standardized questionnaire including a range of demographic factors, medical history, and health-related behaviors (especially smoking exposure status) according to the standardized protocol.

### Measurement of smoking and covariates

The categories of educational attainment included 0–6 years (primary school or less), 6–12 years (middle school to high school or the equivalent), and ≥13 years (completed a university or other tertiary education). The occupation types were classified into the following three categories: white collar (professional, government), light physical labor (skilled worker, service, merchant), and hard physical labor (famer and worker in factories, manufacturing and transportation). Marital status was classified into the following two categories: married (living as a couple) and single/divorced/widowed (not living with a spouse). The categories of smoking status consisted of never smoker, former smoker, and current smoker. A current smoker was defined as a person who, at the time of the survey, smoked a tobacco product. A former smoker was defined as a person who had smoked daily for at least 6 months during their lives but at the time of the survey did not use a tobacco product [29]. A passive smoker was defined as a person who was exposed to smoke exhaled by a smoker for more than 15 minutes per day more than once per week [29].

The information regarding cigarettes per day (CPD), age of smoking initiation (SI), years of smoking, and places and sources of passive smoking was self-reported.

### Statistical analysis

SPSS version 19.0 (No. of Serial: 5076595) was used for the data analysis. The significance level for all tests was set at a two-tailed α value of 0.05. The differences in means and proportions were tested using the t-test and chi-square test, respectively. Logistic regression models were used to identify the risk factors for passive smoking from offspring sources.

### Ethical considerations

The committee for medical ethics of the Chinese PLA General Hospital examined and approved our study and before completing the questionnaire, each involved participant signed an informed consent.

## Results

A total of 2,277 participants (943 males and 1,334 females) in 2001 were involved in our study and 2,102 participants (848 males and 1,254 females) in 2010 completed the survey and were involved in our study. The general characteristics of the participants in the two surveys (unadjusted and adjusted) are shown in Table 1. Figures capturing the differences between the first and second measurements of average age (in 2001, it was 67.9 years old, and in 2010, it was 71.2 years old, P<0.001). There were no significant differences between 2001 and 2010 in the distributions of sex or marital status (P = 0.471 and 0.759, respectively), while a difference between 2001 and 2010 measurement in education was apparent (Table 1).

**Table 1:** General characteristics of the participants in two Surveys from 2001 and 2010.

Characteristics	2001 (n = 2,277)	2010 (n = 2,102)	P
Age, mean (SD)	67.9 (5.8)	71.2 (6.6)	<0.001
	% (95% CI)	% (95% CI)	
60-	63.8 (61.8–65.7)	39.1 (37.0–41.2)	<0.001
70-	31.8 (29.9–33.8)	51.7 (49.6–53.9)	
80-	4.4 (3.6–5.2)	9.2 (8.0–10.5)	
Sex			
Male	41.4 (39.4–43.4)	40.3 (38.2–42.4)	0.471
Female	58.6 (56.6–60.6)	59.7 (57.6–61.8)	
Marital status			0.759
Married	84.1 (82.6–85.6)	84.4 (82.8–86.0)	
Single or divorced or widowed	15.9 (14.4–17.5)	15.6 (14.1–17.2)	
Occupation			<0.001
White collar	48.8 (46.7–50.9)	33.7 (31.7–35.8)	
Light physical labor	44.7 (42.7–46.8)	36.2 (34.2–38.3)	
Hard physical labor	6.5 (5.5–7.5)	30.1 (28.1–32.0)	
Education level (years)			<0.001
0–6	42.3 (40.3–44.3)	27.6 (25.9–29.5)	
7–12	34.8 (32.9–36.8)	40.4 (38.3–42.5)	
13∼	22.9 (21.2–24.6)	32.0 (30.0–34.0)	

*P is for 2001 vs*. *2010;*

*Data are mean (SD) for continuous values or n (%) for category values*.

### Prevalence of active smoking

The prevalence of current active smoking among males and females by selected characteristics in 2001 and 2010 is shown in Table 2. The prevalence of current smoking was 15.4% (95% CI: 13.9–16.9%) in 2001 and decreased to 11.0% (95% CI: 9.7–12.3%) in 2010 (P < 0.001). The current smoking prevalence decreased by 3.5% (24.7 vs. 21.2%, P = 0.081) in males, while in females, the prevalence decreased by nearly half (from 8.8% to 4.1%) (P<0.001). The prevalence of current smoking declined as age increased; this trend was more evident in 2010 than in 2001. In particular, 60–70 year old males had the highest prevalence (27.2% in 2001 and 30.7% in 2010) of current smoking in both surveys. The prevalence of current smoking declined dramatically in the light physical labor group in both males and females; the prevalence changed from 35.0% (95% CI: 28.9–41.0%) in 2001 to 15.7% (95% CI: 12.0–19.4%) in 2010 among males, and it decreased by 8.3% among females (P<0.001). In both surveys, the prevalence of current smoking was significantly lower among those with higher educational attainment.

**Table 2:** Prevalence (95% CI) of current active smoking among males and females by selected characteristics (2001–2010).

	Total			Male			Female		
Characteristics	2001 survey	2010 survey	P1	2001 survey	2010 survey	P1	2001 survey	2010 survey	P1
	(n = 2,277)	(n = 2,102)		(n = 943)	(n = 848)		(n = 1,334)	(n = 1,254)	
	% (95% CI)	% (95% CI)		% (95% CI)	% (95% CI)		% (95% CI)	% (95% CI)	
Total	15.4 (13.9–16.9)	11.0 (9.7–12.3)	**<0.001**	24.7 (22.0–27.5)	21.2 (18.5–24.0)	0.081	8.8 (7.3–10.3)	4.1 (3.0–5.2)	**<0.001**
Age group									
60-	15.9 (14.0–17.8)	12.8 (10.5–15.1)	**0.044**	27.2 (23.6–30.8)	30.7 (25.3–36.2)	0.274	8.1 (6.2–9.9)	3.4 (1.8–4.9)	**<0.001**
70-	15.2 (12.6–17.8)	10.2 (8.4–12.0)	**0.002**	22.7 (18.0–27.4)	18.0 (14.4–21.6)	0.115	9.6 (6.8–12.5)	4.7 (3.1–6.4)	**0.002**
80-	9.0 (3.3–14.7)	7.7 (3.9–11.5)	0.707	2.6 (0.0–8.0)	10.4 (4.8–16.1)	0.135	12.9 (4.3–21.5)	3.8 (0.0–8.1)	**0.045**
P2	0.177	0.064		**0.002**	**<0.001**		0.327	0.495	
Marital status									
Married	15.5 (13.8–17.1)	11.3 (9.8–12.8)	**<0.001**	24.8 (22.0–27.7)	21.3 (18.4–24.1)	0.085	7.6 (6.0–9.2)	3.4 (2.3–4.6)	**<0.001**
Widowed or divorced	14.9 (11.2–18.6)	9.5 (6.3–12.6)	**0.030**	23.2 (13.0–33.4)	20.9 (10.9–30.9)	0.837	12.9 (9.1–16.8)	6.5 (3.5–9.5)	**0.012**
P2	0.775	0.332		0.761	0.945		**0.004**	**0.025**	
Occupation									
White collar	13.0 (11.0–15.0)	9.5 (7.3–11.6)	**0.02**	19.9 (16.8–23.1)	20.6 (15.9–25.2)	0.821	4.5 (2.6–6.3)	1.7 (0.4–2.9)	**0.017**
Light physical labor	17.0 (14.7–19.3)	9.3 (7.3–11.4)	**<0.001**	35.0 (28.9–41.0)	15.7 (12.0–19.4)	**<0.001**	11.4 (9.1–13.6)	3.1 (1.4–4.9)	**<0.001**
Hard physical labor	21.6 (14.9–28.3)	14.7 (12.0–17.5)	**0.039**	30.5 (20.3–40.7)	33.9 (26.9–40.9)	0.587	10.6 (3.0–18.2)	7.1 (4.7–9.5)	0.31
P2	**0.004**	**0.002**		**<0.001**	**<0.001**		**<0.001**	**<0.001**	
Education level (years)									
0–6	17.9 (15.4–20.3)	12.6 (9.9–15.3)	**0.006**	32.1 (26.3–37.8)	24.3 (17.3–31.3)	0.099	12.6 (10.2–15.1)	8.6 (5.9–11.2)	**0.034**
7–12	15.0 (12.5–17.5)	10.7 (8.6–12.8)	**0.009**	27.2 (22.5–31.9)	26.9 (21.8–32.0)	0.921	5.4 (3.3–7.5)	2.2 (1.0–3.4)	**0.006**
13∼	11.3 (8.6–14.1)	10.0 (7.7–12.2)	0.445	16.4 (12.4–20.4)	16.0 (12.4–19.6)	0.881	2.2 (0.1–4.3)	0.8 (0.0–1.8)	0.199
P2	**0.004**	0.315		**<0.001**	**0.001**		**<0.001**	**<0.001**	

*P1 is for 2001 vs*. *2010;*

*P2 is for the comparison of characteristics groups*.

The percentage of former smokers was 16.4% (95% CI: 14.9–18.0%) in 2001 and 16.2% (95% CI: 14.6–17.8%) in 2010. There was no significant change in either gender in the surveys; in males, the rate of former smoking changed by 0.8% (P = 0.731), while in females, the rate changed by 0.6% (P = 0.471).

The proportion that never smoked was 68.2% (95% CI: 66.3–70.1%) in 2001 and 72.8% (95% CI: 70.9–74.7%) in 2010 (P<0.05). The proportion increased significantly in females (86.6 vs. 90.8%, P = 0.001), but the change was not significant in males (42.1 vs. 46.3%, P = 0.071).

### Prevalence of passive smoking

The prevalence of passive smoking among males and females by selected characteristics in 2001 and 2010 is shown in Table 3. The overall prevalence of passive smoking was 30.5% (95% CI: 28.6–32.4%) in 2001, which decreased slightly to 30.0% (95% CI: 28.1–32.0%) in 2010 (P = 0.717). The prevalence of passive smoking changed slightly in both sexes (P = 0.399 and 0.233, respectively). The prevalence of passive smoking declined with increasing age among males in 2001 and females in 2010. Females 60- to 70 years old had the highest prevalence (40.5% in 2001 and 41.6% in 2010) of passive smoking in both surveys. Interestingly, the prevalence of passive smoking increased by 10.5% among 70- to 80-year-old males (P<0.001). The prevalence of passive smoking had the greatest increase (increased by 15.2%, P = 0.004) in the heavy physical labor group among males and the greatest decrease (decreased by 7.3%, P = 0.018) in the light labor group among females. The prevalence of passive smoking was significantly lower among those with a higher education level in both sexes in 2010 and in females in 2001 (Table 3).

**Table 3:** Prevalence (95% CI) of passive smoking among males and females by selected characteristics (2001–2010).

	Total			Male			Female		
Characteristics	2001 survey	2010 survey	P1	2001 survey	2010 survey	P1	2001 survey	2010 survey	P1
	(n = 2,277)	(n = 2,102)		(n = 943)	(n = 848)		(n = 1,334)	(n = 1,254)	
	% (95% CI)	% (95% CI)		% (95% CI)	% (95% CI)		% (95% CI)	% (95% CI)	
Total	30.5 (28.6–32.4)	30.0 (28.1–32.0)	0.717	19.6 (17.1–22.2)	21.2 (18.5–24.0)	0.399	38.2 (35.6–40.8)	36.0 (33.3–38.6)	0.233
Age group									
60-	33.5 (31.0–35.9)	33.6 (30.4–36.9)	0.943	23.3 (19.9–26.7)	18.4 (13.8–22.9)	0.097	40.5 (37.2–43.8)	41.6 (37.5–45.8)	0.685
70-	25.1 (21.9–28.3)	28.7 (26.0–31.4)	0.092	13.3 (9.5–17.1)	23.8 (19.8–27.7)	**<0.001**	33.9 (29.3–38.5)	32.2 (28.5–35.8)	0.563
80-	27.0 (18.2–35.9)	22.2 (16.3–28.1)	0.356	13.2 (1.9–24.4)	18.3 (11.1–25.4)	0.468	35.5 (23.2–47.7)	27.9 (17.7–38.0)	0.331
P2	**<0.001**	**0.003**		**0.001**	0.155		0.066	**0.001**	
Marital status									
Married	31.1 (29.0–33.2)	29.7 (27.5–31.8)	0.343	20.0 (17.4–22.7)	20.5 (17.7–23.3)	0.815	40.4 (37.4–43.4)	36.9 (33.9–39.9)	0.103
Widowed or divorced	27.6 (22.9–32.2)	32.0 (26.9–37.1)	0.200	14.5 (6.0–23.0)	29.9 (18.6–41.1)	**0.031**	30.6 (25.3–35.9)	32.6 (26.8–38.3)	0.621
P2	0.180	0.391		0.265	0.072		**0.002**	0.199	
Occupation									
White collar	24.9 (22.4–27.5)	26.7 (23.4–29.9)	0.411	20.6 (17.4–23.7)	21.6 (16.8–26.3)	0.722	30.4 (26.4–34.5)	30.2 (25.8–34.6)	0.945
Light physical labor	37.9 (34.9–40.9)	27.7 (24.5–30.9)	**<0.001**	20.6 (15.5–25.7)	19.2 (15.2–23.1)	0.663	43.4 (39.9–46.9)	36.1 (31.3–40.9)	**0.018**
Hard physical labor	21.6 (14.9–28.3)	36.6 (32.8–40.3)	**0.001**	9.8 (3.2–16.3)	25.0 (18.6–31.4)	**0.004**	36.4 (24.5–48.3)	41.2 (36.6–45.7)	0.459
P2	**<0.001**	**<0.001**		0.063	0.283		**<0.001**	**0.004**	
Education level (years)									
0–6	35.5 (32.5–38.5)	37.6 (33.6–41.5)	0.412	16.6 (12.0–21.2)	32.4 (24.8–40.1)	**<0.001**	42.5 (38.8–46.1)	39.4 (34.7–44.0)	0.300
7–12	28.8 (25.6–31.9)	31.2 (28.1–34.3)	0.277	20.9 (16.6–25.2)	20.1 (15.5–24.7)	0.791	34.9 (30.5–39.4)	37.1 (33.1–41.2)	0.471
13∼	24.0 (20.3–27.7)	22.0 (18.9–25.1)	0.414	20.6 (16.2–25.0)	18.0 (14.2–21.7)	0.368	30.1 (23.5–36.8)	28.1 (22.7–33.5)	0.641
P2	**<0.001**	**<0.001**		0.355	**0.001**		**0.002**	**0.008**	

*P1 is for 2001 vs*. *2010;*

*P2 is for the comparison of characteristics groups*.

### Patterns of tobacco exposure in elderly populations

Table 4 shows the pattern of tobacco exposure among participants in the 2001 and 2010 surveys. CPD changed slightly in both surveys. The most significant occurrence of passive smoking was with family, and the most serious passive smoking exposure occurred among women at home, which was approximately 20% higher than that among men at home (P<0.001 in both measurements), and the prevalence changed slightly (2.4%, P = 0.215) between the two surveys. However, the main source of passive smoking in females changed between surveys. Differences between the first and second measurements indicate that passive smoking resulting from exposure to a smoking spouse decreased by nearly half (13.0%, P<0.001) and that passive smoking exposure resulting from smoking offspring increased 2-fold (P<0.001). This trend was observed in males as well; passive smoking exposure from a spouse decreased from 5.7 to 2.4% while passive smoking exposure from offspring increased from 7.3 to 14.5% (P<0.001) (Table 4).

**Table 4:** Smoking status of the participants of two Surveys in 2001 and 2010.

	Total			Male			Female		
Mean(SD)	2001 (n = 2,277)	2010 (n = 2,102)	P	2001 (n = 943)	2010 (n = 848)	P	2001 (n = 1,334)	2010 (n = 1,254)	P
CPD	13.3 (9.2)	13.0 (10.2)	0.645	14.8 (9.2)	13.9 (10.2)	0.131	8.6 (7.4)	9.7 (9.8)	0.275
SI	22.1 (9.6)	25.0 (9.4)	**<0.001**	22.1 (8.7)	24.3 (8.2)	**<0.001**	21.8 (12.0)	27.5 (12.7)	**<0.001**
Lasting time	34.3 (15.2)	36.8 (14.6)	**0.003**	34.7 (14.1)	37.2 (13.9)	**0.005**	33.1 (18.0)	35.4 (17.0)	0.277
CPD (P)	11.7 (8.3)	10.5 (10.0)	**0.016**	10.1 (7.9)	9.6 (8.1)	0.551	12.4 (8.4)	10.9 (10.6)	**0.020**
Lasting time (P)	25.8 (15.3)	27.2 (15.5)	0.092	17.8 (14.7)	20.7 (14.0)	0.052	28.7 (14.4)	29.8 (15.3)	0.243
% (95% CI)									
Active smoking status									
current	15.4 (13.9–16.9)	11.0 (9.7–12.3)	**<0.001**	24.7 (22.0–27.5)	21.2 (18.5–24.0)	0.081	8.8 (7.3–10.3)	4.1 (3.0–5.2)	**<0.001**
former	16.4 (14.9–18.0)	16.2 (14.6–17.8)	0.823	33.2 (30.2–36.2)	32.4 (29.3–35.6)	0.731	4.6 (3.5–5.7)	5.2 (4.0–6.4)	0.471
Never	68.2 (66.3–70.1)	72.8 (70.9–74.7)	**0.001**	42.1 (38.9–45.3)	46.3 (43.0–49.7)	0.071	86.6 (84.8–88.5)	90.8 (89.1–92.4)	**0.001**
Passive smoking	30.5 (28.6–32.4)	30.0 (28.1–32.0)	0.717	19.6 (17.1–22.2)	21.2 (18.5–24.0)	0.399	38.2 (35.6–40.8)	36.0 (33.3–38.6)	0.233
Places									
Family	26.6 (24.8–28.4)	26.9 (25.0–28.8)	0.817	13.2 (11.0–15.3)	16.8 (14.2–19.3)	0.033	36.1 (33.5–38.6)	33.7 (31.1–36.4)	0.215
Work	2.6 (1.9–3.2)	2.1 (1.5–2.7)	0.320	5.2 (3.8–6.6)	3.2 (2.0–4.4)	0.035	0.7 (0.2–1.1)	1.4 (0.7–2.0)	0.083
Public	1.4 (0.9–1.9)	1.1 (0.6–1.5)	0.283	1.3 (0.6–2.0)	1.3 (0.5–2.1)	0.963	1.5 (0.9–2.2)	0.9 (0.4–1.4)	0.146
Source									
Couple	20.3 (18.6–21.9)	11.5 (10.1–12.8)	**<0.001**	5.7 (4.2–7.2)	2.4 (1.3–3.4)	**<0.001**	30.6 (28.1–33.1)	17.6 (15.5–19.7)	**<0.001**
Off-springs	6.2 (5.2–7.1)	15.0 (13.5–16.6)	**<0.001**	7.3 (5.7–9.0)	14.5 (12.1–16.9)	**<0.001**	5.3 (4.1–6.5)	15.4 (13.4–17.4)	**<0.001**
colleagues	2.6 (1.9–3.2)	2.6 (1.9–3.3)	0.964	5.3 (3.9–6.7)	3.7 (2.4–4.9)	0.094	0.6 (0.2–1.0)	1.8 (1.1–2.6)	**0.004**
others	1.5 (1.0–2.0)	1.0 (0.5–1.4)	0.082	1.3 (0.6–2.0)	0.7 (0.1–1.3)	0.231	1.7 (1.0–2.4)	1.1 (0.5–1.7)	0.193

P is for 2001 vs. 2010

Data are mean (SD) for continuous values or % (95% CI) for category value

CPD = cigarettes per day

SI = age of smoking initiation

CPD (P) = cigarettes per day of passive smoking.

We found the same change in the 835 participants who were interviewed in both 2001 and 2010 surveys (Table 5).

**Table 5:** Smoking status of the participants.

	Male (n = 288)			Female (n = 547)		
Mean(SD)	2001	2010	P	2001	2010	P
CPD	13.1 (8.3)	13.0 (9.4)	0.771	9.4 (9.2)	9.7 (8.3)	0.792
SI	24.0 (9.1)	25.4 (9.4)	**0.017**	24.7 (10.0)	26.2 (10.4)	0.140
Years of smoking	33.6 (11.8)	37.3 (14.3)	**<0.001**	31.8 (13.0)	37.0 (14.6)	**0.001**
CPD (P)	12.5 (7.5)	9.5 (8.3)	0.182	12.7 (7.8)	13.3 (13.6)	0.643
Years of passive smoking	19.6 (11.7)	22.8 (13.3)	0.164	30.4 (13.7)	35.3 (15.7)	**0.002**
% (95% CI)						
Active smoking status			**<0.001**			**<0.001**
Current	26.0 (20.9–31.1)	16.3 (12.0–20.6)	**<0.001**	7.9 (5.6–10.1)	4.0 (2.4–5.7)	**<0.001**
Former	30.9 (25.5–36.3)	40.6 (34.9–46.3)	**<0.001**	4.8 (3.0–6.5)	9.1 (6.7–11.6)	**<0.001**
Never	43.1 (37.3–48.8)	43.1 (37.3–48.8)	0.918	87.4 (84.6–90.2)	86.8 (84.0–89.7)	0.936
Current smoking prevalence						
60–69	28.2 (22.2–34.2)	18.6 (13.5–23.8)	**<0.001**	7.7 (5.3–10.0)	3.9 (2.2–5.7)	**<0.001**
70–79	20.3 (10.2–30.4)	9.4 (2.0–16.7)	**<0.001**	8.1 (1.1–15.0)	4.8 (0.6–10.3)	**0.015**
≥80	0 (0.0)	0 (0.0)		1 (50.0)	0 (0.0)	
Passive smoking	18.4 (13.9–22.9)	22.6 (17.7–27.4)	**<0.001**	35.8 (31.8–39.9)	35.1 (31.1–39.1)	**<0.001**
60–69	20.9 (15.5–26.3)	24.6 (18.8–30.3)	**<0.001**	36.4 (32.1–40.8)	35.0 (30.7–39.3)	**<0.001**
70–79	10.9 (3.1–18.8)	17.2 (7.7–26.7)	0.091	29.0 (17.4–40.7)	33.9 (21.8–46.0)	**0.037**
≥80	0 (0.0)	0 (0.0)		2 (100.0)	2 (100.0)	
Places			**<0.001**			**<0.001**
Family	12.5 (8.7–16.3)	18.8 (14.2–23.3)	**<0.001**	34.6 (30.6–38.6)	33.1 (29.1–37.1)	**<0.001**
Work	5.6 (2.9–8.2)	2.8 (0.9–4.7)	0.487	1.1 (0.2–2.0)	1.1 (0.2–2.0)	0.795
Public	0.4 (0.3–1.0)	0.7 (0.3–1.7)	0.933	0.2 (0.1–0.5)	0.9 (0.1–1.7)	0.923
Source			**<0.001**			**<0.001**
Couple	5.6 (2.9–8.2)	2.8 (0.9–4.7)	**<0.001**	28.9 (25.1–32.7)	14.6 (11.7–17.6)	**<0.001**
Off-springs	6.6 (3.7–9.5)	16.7 (12.3–21.0)	**<0.001**	4.4 (2.7–6.1)	17.7 (14.5–20.9)	**<0.001**
Colleagues	5.2 (2.6–7.8)	3.1 (1.1–5.2)	0.475	1.1 (0.2–2.0)	1.7 (0.6–2.7)	0.750
Others	1.0 (0.1–2.2)	0 (0.0)		1.5 (0.5–2.5)	0.9 (0.1–1.7)	0.784

P is for 2001 vs. 2010

Data are mean (SD) for continuous values or % (95% CI) for category value

CPD = cigarettes per day

SI = age of smoking initiation.

We fit logistic regression models to examine the risk characteristics of participants exposed to the secondhand smoke of offspring. The results are shown in Table 6. After adjustment, the risk of being exposed to the secondhand smoke of offspring did not have a significant difference between males and females (P = 0.962). The risk of being exposed to the secondhand smoke of offspring increased among elderly not living as a couple (OR = 1.52, 95% CI: 1.10–2.08) compared with those living with a spouse after adjustment. The risk also increased among elderly in the light labor (OR = 1.45, 95% CI: 1.05–2.00) and heavy labor (OR = 1.35, 95% CI: 0.94–1.95) categories of work compared to those participants classified as white collar. And a significant trend was observed with education level (P<0.05).

**Table 6:** Odds ratio (OR) of offspring source of passive smoking (123 male and 193 female) vs. non-passive smoking (668 male and 803 female) according to selected characteristics in 2010.

	Crude	Adjusted
Sex		
Male	1	1
Female	**1.31 (1.02–1.67)**	0.96 (0.73–1.26)
Age (years)		
60-	1	1
70-	1.22 (0.94–1.59)	0.98 (0.74–1.30)
80-	0.89 (0.56–1.41)	0.71 (0.44–1.16)
P for trend	0.706	0.293
Marital status		
Married	1	1
Widowed or divorced	**1.77 (1.32–2.38)**	**1.52 (1.10–2.08)**
Occupation		
White collar	1	1
Light physical labor	**1.63 (1.19–2.23)**	**1.45 (1.05–2.00)**
Hard physical labor	**2.16 (1.57–2.98)**	1.35 (0.94–1.95)
P for trend	**<0.001**	0.108
Education level (years)		
0–6	1	1
7–12	**0.52 (0.39–0.69)**	**0.58 (0.43–0.80)**
13∼	**0.36 (0.26–0.49)**	**0.42 (0.29–0.63)**
P for trend	**<0.001**	**<0.001**

Adjusted model involved gender, age group, marital status (married, single or divorced or widowed), occupation (white collar, light physical labor, hard physical labor) and education level (years: ≤6, 7–12, ≥13).

To obtain better external validity, we used the distributions of age and sex in the Beijing urban population in 2001 and 2010 [30] as references, respectively, and adjusted our data; the results were similar (S1–S3 Tables, S1 Table shows the adjusted prevalence of current active smoking among males and females by selected characteristics (gender, age, marriage, education and occupation) in 2001 and 2010; S2 Table shows the adjusted prevalence of passive smoking among males and females by selected characteristics in 2001 and 2010; S3 Table shows the pattern of active smoke exposure (CPD, SI and years of active smoke) and passive smoke exposure (CPD (P), years of passive smoke, the places and sources of passive smoking) in the 2001 and 2010 surveys).

## Discussion

Smoke exposure among the elderly population is an important and potentially preventable health problem. However, to date, few epidemiological studies have assessed the changes and patterns of smoke exposure among the elderly. To the best of our knowledge, this is the first study to assess the changes and patterns of smoking exposure (active and passive) among elderly populations in the past decade in China.

Few studies have specifically examined the prevalence of smoking in elderly populations, while other surveys have mentioned the current smoking prevalence in the elderly; all of these studies included the elderly in the total adult sample and used questionnaires tailored to the adult population. In Europe, the overall smoking prevalence in the elderly (≥65 years old) was 11.5% (15.3% in males and 8.6% in females) in 2010 [31]. In Seongnam, Korea, the overall smoking prevalence in the elderly (≥65 years old) was 11.9% (23.3% in males and 3.9% in females) in 2005[32]. A meta-analysis combining 48 studies showed that the overall prevalence of tobacco use was 13% in both genders (22% in males and 8% in females) among 140,058 elderly subjects (≥60 years old) [33]. In elderly Mexican Americans, the prevalence of smoking in males (ages 65–74) decreased from 41.2 to 19.6% between 1983–1984 and 1993–1994, and in females, the prevalence of smoking decreased from 19.2 to 9.8% [34]. For the Chinese population, a study excluding participants who were 70 years old and older showed that 52% of males were current smokers in 2002 [35]. In our study, we observed that the current smoking prevalence in the elderly population was 15.4% in 2001, and this decreased to 11.0% in 2010. The prevalence remained stable among males (24.7% in 2001 and 21.2% in 2010); although in females, the prevalence decreased significantly from 8.8 to 4.1%, which is similar to the results of the study in Seongnam, Korea in 2005. Although the prevalence in elderly males was lower than in adults overall [35], the prevalence remained high and stable over the past decade. The 60–70-year-old males had the highest prevalence of current smoking in both surveys. In females, for cultural and social reasons[36], the current smoking prevalence was always much lower than that in males, and in this study, it decreased significantly over the past decade.

In our study, we observed an inverse relationship between education level and prevalence of current smoking in both genders over the past decade. A model of the tobacco epidemic was proposed by Lopez and colleagues in 1994 that identified a direct relationship between education level and smoking prevalence in the 1970s, a null relationship in the 1980s and an inverse relationship in the 1990s among elderly populations; among all adults, the relationship between education level and smoking prevalence was slightly inverse in the 1970s, stronger in the 1980s and the strongest in the 1990s [37]. According to this model, the elderly population in our study is in a further stage of the tobacco epidemic.

Compared with the results of the 2002 national tobacco survey, in which average consumption was 15 cigarettes per day among Chinese adult smoking males, [35], the cigarette consumption among elderly smoking males was comparable in 2001 (13.3 cigarettes per day) and decreased slightly in 2010 (13.0 cigarettes per day).

Previous studies have shown that 25% of U.S. children were exposed to passive smoking in 2000 [38]; in Italy, 33% of the elderly population (≥65 years old) was exposed to indoor passive smoking [39]. This means that passive smoking is epidemic throughout the world and affects the health of vulnerable groups. The 2002 national tobacco survey of China showed that the prevalence of passive smoking was 49.7% in urban areas in 2002 [22]. In our study, the prevalence of passive smoking was 30.5% in 2001 and 30.0% in 2010, which was lower than the national prevalence in 2002; this may be due to the higher socio-economic status of our participants from urban Beijing compared to others in China, as higher socio-economic status tends to lead to lower passive smoke exposure [16,40].

In our study, we found a significant decrease in both the prevalence of current smoking and passive smoking among the light physical labor group from 2001 to 2010. This may be related to the step-by-step implementation of a national smoking ban in public places and the ban of tobacco advertising [41]. Further, a similar decrease was found in the middle education group, indicating that the tobacco control program in large cities in China is to some extend influencing citizens of middle socio-economic status.

In our study, we observed a transition of the main source of passive smoking exposure among the elderly in China. Due to cultural and social reasons, most adults were particularly concerned about the health of their children and consciously avoided smoking in front of children, whereas they did not avoid smoking in front of the elderly. In 2010, offspring become the main source of passive smoking exposure among the elderly. Today, there are more than a few adults are living with their parents because having four generations under one roof is an auspicious situation in Chinese culture, and traditionally, supporting the elderly takes the form of family support as its main presentation. The tobacco control frame only emphasizes protecting children and adolescents from secondhand smoking. According to our study, in China, there should be a greater focus on the epidemic of passive smoking exposure in the elderly population, and more specific health education and smoking cessation services should be provided to adults who are living with their parents. Additionally, elderly not living as a couple, the elderly with low education levels and those involved in heavy physical labor work had a higher risk of the secondhand smoke exposure from offspring.

To assure the quality of our study, we used strictly trained interviewers and the standardized protocol. Further, Wanshoulu District is a miniature of Beijing urban area [28]. Furthermore, we used respective percentages of age and gender in Beijing in 2001 and 2010 as a reference and standardized our data to improve the representativeness in S1–S3 Tables, and the results were similar. The response rate was high. Consequently, the prevalence of and changes in smoking exposure (active and passive) in the elderly population could be a miniature of urban areas of Beijing, China. Furthermore, to the best of our knowledge, this is the first study to assess the changes in and patterns of smoking exposure (active and passive) among elderly populations over the past decade in China. However, our study cannot represent the characteristics of rural areas and regions of China with other levels of socio-economic status in which there are more elderly people living without offspring.

Admittedly, this study was a cross-sectional study with two time points conducted in Wanshoulu district (a miniature of Beijing urban area). For feasibility and due to limited financial resources reasons, we used the randomized cluster sampling method, instead of random sampling, and when we calculated the sample size, we multiplied by 1.5 as a cluster effect to improve the representativeness of the sample. Further, unlike a cohort study, this study could only calculate the prevalence of smoking exposure in the two surveys, but it could not predict the incidence accurately. However, approximately 39% (835 participants) of the population was included in both surveys. To be more complete, we conducted the same analyses for the 835 participants included in both surveys. The similar results that we obtained confirmed the changes of smoking exposure in the elderly. Another limitation of our study is the potential information bias inherent in a cross-sectional study, as the information was collected by questionnaires, and the exposure information was all self-reported. However, we used certain definitions to distinguish different smoking exposure statuses, and the interviewers were strictly trained to conduct the face-to-face interview to avoid information bias.

In summary, our study examined the smoke exposure status specifically in the elderly population, and we found that the current smoking prevalence remained high and stable in elderly males, while the prevalence decreased significantly in females. According to the inverse relationship that existed between education level and the prevalence of current smoking, the elderly people in our study were in a further stage of the tobacco epidemic. Additionally, we found a significantly decreased exposure in the light labor group, which might be related to workplace smoking bans in Beijing.

More notably, we observed that passive smoking exposure was epidemic in the elderly population, and offspring have become the main source of passive smoking exposure for the elderly. The elderly not living as couples, those with low education levels and those involved in heavy physical labor have a higher risk of exposure to the secondhand smoke of offspring. According to our study, in China, there should be a greater focus on the epidemic of passive smoking exposure in the elderly population, and more specific health education and smoking cessation services should be provided to adults who are living with their parents. Further, the tobacco control frame should increase focus on the secondhand smoke exposure among elderly. In addition, further studies are needed to assess the conditions among the elderly populations in rural areas of China and in regions with other levels of socio-economic status.

## Supporting Information

S1 Dataset The dataset of the participants in two Surveys from 2001 and 2010. It contains the raw data of 4,379 participants (2,277 of 2001 and 2,102 of 2010) with 24 variables. Including code, gender (male or female), the year of investigation (2001 or 2010), weight, age group (60-, 70- and 80-), education years (0–6, 7–12, ≥13 years), marriage status (married, single or divorced or widowed), occupation (white collar, light physical labor, hard physical labor), CPD (cigarettes per day), SI (age of smoking initiation), years of active smoke, PCPD (cigarettes per day of passive smoking), years of passive smoke, never smoker (yes or no), former smoker (yes or no), current smoker (yes or no), passive smoker (yes or no), passive smoke at home (yes or no), passive smoke in work place (yes or no), passive smoke in public places (yes or no), passive smoke from couple (yes or no), from offspring (yes or no), from colleague (yes or no) and from others (yes or no).
(ZIP)

S1 Table Adjusted prevalence (95%CI) of current active smoking among males and females by selected characteristics (2001–2010). S1 Table shows the adjusted prevalence of current active smoking among males and females by selected characteristics (gender, age, marriage, education and occupation) in 2001 and 2010. P1 is for 2001 vs. 2010; P2 is for the comparison of characteristics groups.
(DOC)

S2 Table Adjusted prevalence (95%CI) of passive smoking among males and females by selected characteristics (2001–2010). S2 Table shows the adjusted prevalence of passive smoking among males and females by selected characteristics (gender, age, marriage, education and occupation) in 2001 and 2010. P1 is for 2001 vs. 2010; P2 is for the comparison of characteristics groups.
(DOC)

S3 Table Adjusted smoking status in the participants of two surveys in 2001 and 2010. S3 Table shows the pattern of active smoke exposure (CPD, SI and years of active smoke) and passive smoke exposure (CPD (P), years of passive smoke, the places and sources of passive smoking) in the 2001 and 2010 surveys; P is for 2001 vs. 2010. Data are mean (SD) for continuous values or % (95%CI) for category value; CPD = cigarettes per day; SI = age of smoking initiation; CPD (P) = cigarettes per day of passive smoking.
(DOC)

## References

[1] US Department of Health Human Services (2014) The health consequences of smoking—50 years of progress: A report of the surgeon general Atlanta, GA: US Department of Health and Human Services, Centers for Disease Control and Prevention, National Center for Chronic Disease Prevention and Health Promotion, Office on Smoking and Health 17. 24455788

[2] Lam TH (2012) Absolute risk of tobacco deaths: one in two smokers will be killed by smoking: comment on "Smoking and all-cause mortality in older people". Arch Intern Med 172: 845–846. 10.1001/archinternmed.2012.1927 22688993

[3] Watson WL, Conte AJ (1954) Smoking and lung cancer. Cancer 7: 245–249. 13141215 10.1002/1097-0142(195403)7:2<245::aid-cncr2820070206>3.0.co;2-v

[4] Stell P (1972) Smoking and laryngeal cancer. The Lancet 299: 617–618. 10.1016/s0140-6736(72)90411-4 4110316

[5] Michalopoulos A (2005) Smoking and COPD. Tobacco Induced Diseases 3: 30.

[6] Tresch DD, Aronow WS (1996) Smoking and coronary artery disease. Clinics in geriatric medicine 12: 23–32. 8653660

[7] Shinton R, Beevers G (1989) Meta-analysis of relation between cigarette smoking and stroke. BMJ: British Medical Journal 298: 789 2496858 10.1136/bmj.298.6676.789 PMC1836102

[8] Rimm EB, Chan J, Stampfer MJ, Colditz GA, Willett WC (1995) Prospective study of cigarette smoking, alcohol use, and the risk of diabetes in men. Bmj 310: 555–559. 7888928 10.1136/bmj.310.6979.555 PMC2548937

[9] Willi C, Bodenmann P, Ghali WA, Faris PD, Cornuz J (2007) Active smoking and the risk of type 2 diabetes: a systematic review and meta-analysis. JAMA 298: 2654–2664. 18073361 10.1001/jama.298.22.2654

[10] He Y (1989) [Women's passive smoking and coronary heart disease]. Zhonghua Yu Fang Yi Xue Za Zhi 23: 19–22. 2731453

[11] He Y, Lam TH, Jiang B, Wang J, Sai X, Fan L, et al (2008) Passive smoking and risk of peripheral arterial disease and ischemic stroke in Chinese women who never smoked. Circulation 118: 1535–1540. 10.1161/CIRCULATIONAHA.108.784801 18809795

[12] Nishino Y, Tsuji I, Tanaka H, Nakayama T, Nakatsuka H, Ito H, et al. (2014) Stroke mortality associated with environmental tobacco smoke among never-smoking Japanese women: A prospective cohort study. Prev Med. 10.1016/j.ypmed.2014.06.029 24983889

[13] Hagstad S, Bjerg A, Ekerljung L, Backman H, Lindberg A, Ronmark E, et al (2014) Passive smoking exposure is associated with increased Risk of COPD in never smokers. Chest 145: 1298–1304. 10.1378/chest.13-1349 24356778

[14] He Y, Jiang B, Li LS, Li LS, Ko L, Wu L, et al (2012) Secondhand smoke exposure predicted COPD and other tobacco-related mortality in a 17-year cohort study in China. Chest 142: 909–918. 22628493 10.1378/chest.11-2884

[15] Park S, Jee SH, Shin HR, Park EH, Shin A, Jung KW, et al (2014) Attributable fraction of tobacco smoking on cancer using population-based nationwide cancer incidence and mortality data in Korea. BMC Cancer 14: 406 10.1186/1471-2407-14-406 24902960 PMC4090397

[16] Hovell MF, Hughes SC (2009) The behavioral ecology of secondhand smoke exposure: A pathway to complete tobacco control. Nicotine Tob Res 11: 1,254–1264. 10.1093/ntr/ntp133 PMC2782259 19776346

[17] McNeill A, Guignard R, Beck F, Marteau R, Marteau TM (2014) Understanding increases in smoking prevalence: case study from France in comparison with England 2000–2010. Addiction. 10.1111/add.12789 25393099

[18] Jemal A, Thun M, Yu XQ, Hartman AM, Cokkinides V, Center MM, et al (2011) Changes in smoking prevalence among U.S. adults by state and region: Estimates from the Tobacco Use Supplement to the Current Population Survey, 1992–2007. BMC Public Health 11: 512 10.1186/1471-2458-11-512 21714876 PMC3150264

[19] Nwhator SO (2012) Nigeria's costly complacency and the global tobacco epidemic. J Public Health Policy 33: 16–33. 10.1057/jphp.2011.58 22170505

[20] Gallus S, Lugo A, La Vecchia C, Boffetta P, Chaloupka FJ, Colombo P, et al (2014) Pricing Policies And Control of Tobacco in Europe (PPACTE) project: cross-national comparison of smoking prevalence in 18 European countries. Eur J Cancer Prev 23: 177–185. 10.1097/CEJ.0000000000000009 24441832

[21] Yang G, Fan L, Tan J, Qi G, Zhang Y, Samet JM, et al (1999) Smoking in China: findings of the 1996 National Prevalence Survey. JAMA 282: 1247–1253. 10517427 10.1001/jama.282.13.1247

[22] Yang GH, Ma JM, Liu N, Zhou LN (2005) [Smoking and passive smoking in Chinese, 2002]. Zhonghua Liu Xing Bing Xue Za Zhi 26: 77–83. 15921604

[23] Yang Y, Wang J-j, Wang C-x, Li Q, Yang G-H (2010) Awareness of tobacco-related health hazards among adults in China. Biomedical and Environmental Sciences 23: 437–444. 10.1016/S0895-3988(11)60004-4 21315241

[24] Katanoda K, Jiang Y, Park S, Lim MK, Qiao YL, Inoue M. (2014) Tobacco control challenges in East Asia: proposals for change in the world's largest epidemic region. Tob Control 23: 359–368. 10.1136/tobaccocontrol-2012-050852 23596197 PMC4078676

[25] Desormais I, Aboyans V, Guerchet M, Ndamba-Bandzouzi B, Mbelesso P, Dantoine T, et al. (2014) Prevalence of peripheral artery disease in the elderly population in urban and rural areas of Central Africa: the EPIDEMCA study. Eur J Prev Cardiol. 10.1177/2047487314557945 25376847

[26] Lam TH, Xu L, Schooling CM, Chan WM, Lee SY, Leung GM. (2014) Smoking and mortality in a prospective cohort study of older Chinese in Hong Kong. Addiction. 10.1111/add.12776 25331629

[27] He Y, Jiang B, Li LS, Li LS, Sun DL, Wu L, et al (2014) Changes in smoking behavior and subsequent mortality risk during a 35-year follow-up of a cohort in Xi'an, China. Am J Epidemiol 179: 1060–1070. 10.1093/aje/kwu011 24674900

[28] He Y, Jiang B, Wang J, Feng K, Chang Q, Fan L, et al (2006) Prevalence of the metabolic syndrome and its relation to cardiovascular disease in an elderly Chinese population. J Am Coll Cardiol 47: 1588–1594. 16630995 10.1016/j.jacc.2005.11.074

[29] World Health Organization (1984) Guidelines for the conduct of tobacco-smoking surveys among health professionals: report of a WHO meeting held in Winnipeg, Canada, 7–9 July 1983 in collaboration with UICC and ACS.

[30] Ministry of health of the people's republic of China. Available: http://wsbmohgovcn/zwgkzt/ptjnj/listshtml.

[31] Lugo A, La Vecchia C, Boccia S, Murisic B, Gallus S (2013) Patterns of smoking prevalence among the elderly in Europe. Int J Environ Res Public Health 10: 4418–4431. 10.3390/ijerph10094418 24048208 PMC3799502

[32] Kim SK, Park JH, Lee JJ, Lee SB, Kim TH, Han JW, et al (2013) Smoking in elderly Koreans: prevalence and factors associated with smoking cessation. Arch Gerontol Geriatr 56: 214–219. 10.1016/j.archger.2012.08.018 22995342

[33] Marinho V, Laks J, Coutinho ES, Blay SL (2010) Tobacco use among the elderly: a systematic review and meta-analysis. Cad Saude Publica 26: 2213–2233. 21243218 10.1590/s0102-311x2010001200002

[34] Markides KS, Miller TQ, Ray LA (1999) Changes in the smoking behavior of elderly Mexican Americans in the Southwest from 1982–1984 to 1993–1994. Prev Med 28: 251–254. 10072742 10.1006/pmed.1998.0411

[35] Yang G, Kong L, Zhao W, Wan X, Zhai Y, Chen LC, et al (2008) Emergence of chronic non-communicable diseases in China. Lancet 372: 1697–1705. 10.1016/S0140-6736(08)61366-5 18930526

[36] Jiang Y, Li Q, Xiao L, Feng GZ, Yang Y, Yang YN. (2011) [Epidemic and control on tobacco in China]. Zhonghua Liu Xing Bing Xue Za Zhi 32: 1181–1187. 22336596

[37] Lopez AD, Collishaw NE, Piha T (1994) A descriptive model of the cigarette epidemic in developed countries. Tobacco control 3: 242.

[38] Soliman S, Pollack HA, Warner KE (2004) Decrease in the prevalence of environmental tobacco smoke exposure in the home during the 1990s in families with children. Am J Public Health 94: 314–320. 14759948 10.2105/ajph.94.2.314 PMC1448249

[39] Simoni M, Jaakkola MS, Carrozzi L, Baldacci S, Di Pede F, Viegi G. (2003) Indoor air pollution and respiratory health in the elderly. Eur Respir J Suppl 40: 15s–20s. 12762569 10.1183/09031936.03.00403603

[40] Zhang DM, Hu Z, Orton S, Wang JJ, Zheng JZ, Qin X, et al (2013) Socio-economic and psychosocial determinants of smoking and passive smoking in older adults. Biomed Environ Sci 26: 453–467. 10.3967/0895-3988.2013.06.006 23816579

[41] Jha P, Peto R (2014) Global effects of smoking, of quitting, and of taxing tobacco. N Engl J Med 370: 60–68. 10.1056/NEJMra1308383 24382066

