# Supplementary material for: Changes in and Patterns of Smoking Exposure in an Elderly Urban Population in Beijing: 2001–2010
Source: PLoS One. 2015 Mar 18;10(3):e0118500. doi: 10.1371/journal.pone.0118500 (PMC4364981; doi:10.1371/journal.pone.0118500)
Supplement: S1 Table — shows the adjusted prevalence of current active smoking among males and females by selected characteristics (gender, age, marriage, education and occupation) in 2001 and 2010. P1 is for 2001 vs. 2010; P2 is for the comparison of characteristics groups. (DOC) [file pone.0118500.s002.doc]

**S1 Table. Adjusted prevalence (95%CI) of current active smoking among males and females by selected characteristics (2001-2010)**

|  | Total | | | Male | | | Female | | |
| --- | --- | --- | --- | --- | --- | --- | --- | --- | --- |
| Characteristics | 2001 survey (n=2277) | 2010 survey (n=2102) | P1 | 2001 survey (n=943) | 2010 survey (n=848) | P1 | 2001 survey (n=1334) | 2010 survey (n=1254) | P1 |
|  | %(95%CI) | %(95%CI) |  | %(95%CI) | %(95%CI) |  | %(95%CI) | %(95%CI) |  |
| Total | 15.3(13.8-16.7) | 11.8(10.5-13.2) | **0.001** | 24.3(21.5-27.0) | 23.6(20.7-26.5) | 0.740 | 8.9(7.4-10.5) | 3.9(2.8-5.0) | **<0.001** |
| Age group |  |  |  |  |  |  |  |  |  |
| 60- | 16.2(14.2-18.1) | 14.4(12.3-16.5) | 0.222 | 27.2(23.6-30.8) | 30.7(26.4-35.1) | 0.209 | 8.1(6.2-9.9) | 3.3(2.0-4.7) | **<0.001** |
| 70- | 14.9(12.3-17.6) | 9.9(7.8-12.1) | **0.004** | 22.7(17.8-27.6) | 18.0(13.6-22.4) | 0.159 | 9.6(6.8-12.5) | 4.7(2.8-6.7) | **0.005** |
| 80- | 9.3(4.9-13.7) | 6.6(3.6-9.6) | 0.321 | 2.6(1.6-6.8) | 10.4(4.7-16.2) | 0.094 | 12.9(6.5-19.3) | 3.8(0.7-6.9) | **0.008** |
| P2 | 0.064 | **<0.001** |  | **<0.001** | **<0.001** |  | 0.239 | 0.508 |  |
| Marital status |  |  |  |  |  |  |  |  |  |
| Married | 15.4(13.8-17.0) | 12.3(10.8-13.9) | 0.007 | 24.5(21.7-27.4) | 23.6(20.7-26.6) | 0.648 | 7.6(6.0-9.3) | 3.4(2.2-4.5) | **<0.001** |
| Widowed or divorced | 14.6(11.1-18.1) | 9.0(5.8-12.2) | 0.025 | 21.0(11.3-30.6) | 22.8(11.4-34.2) | 0.747 | 13.1(9.4-16.9) | 6.0(3.1-8.9) | **0.004** |
| P2 | 0.659 | 0.094 |  | 0.481 | 0.950 |  | 0.003 | 0.067 |  |
| Occupation |  |  |  |  |  |  |  |  |  |
| White collar | 13.0(11.0-15.0) | 10.4(8.1-12.7) | 0.097 | 19.5(16.4-22.7) | 23.0(18.1-27.9) | 0.217 | 4.7(2.8-6.6) | 1.7(0.4-2.9) | **0.010** |
| Light physical labor | 16.8(14.5-19.1) | 9.9(7.8-12.0) | **<0.001** | 34.5(28.5-40.6) | 17.4(13.6-21.3) | **<0.001** | 11.4(9.1-13.6) | 2.7(1.1-4.2) | **<0.001** |
| Hard physical labor | 21.4(14.7-28.1) | 16.0(13.1-18.9) | 0.127 | 29.7(19.5-39.9) | 38.0(30.7-45.2) | 0.197 | 11.4(3.6-19.3) | 7.2(4.7-9.6) | 0.165 |
| P2 | **0.007** | **0.001** |  | **<0.001** | **<0.001** |  | **<0.001** | **<0.001** |  |
| Education level (years) |  |  |  |  |  |  |  |  |  |
| 0-6 | 17.6(15.2-20.0) | 12.6(9.8-15.5) | **0.013** | 31.3(25.6-37.0) | 26.3(18.2-34.3) | 0.315 | 12.7(10.3-15.1) | 8.6(5.8-11.3) | **0.040** |
| 7-12 | 14.9(12.4-17.4) | 11.6(9.5-13.7) | **0.047** | 26.6(21.9-31.2) | 29.9(24.7-35.1) | 0.364 | 5.5(3.4-7.7) | 2.2(1.0-3.3) | **0.003** |
| 13~ | 11.4(8.6-14.1) | 11.6(9.2-13.9) | 0.969 | 16.4(12.4-20.4) | 18.5(14.8-22.1) | 0.451 | 2.1(0.0-4.2) | 0.7(0.0-1.7) | 0.178 |
| P2 | **0.007** | 0.802 |  | **<0.001** | **0.002** |  | **<0.001** | **<0.001** |  |

*P1 is for 2001 vs. 2010;*

*P2 is for the comparison of characteristics groups.*
