# Supplementary material for: Changes in and Patterns of Smoking Exposure in an Elderly Urban Population in Beijing: 2001–2010
Source: PLoS One. 2015 Mar 18;10(3):e0118500. doi: 10.1371/journal.pone.0118500 (PMC4364981; doi:10.1371/journal.pone.0118500)
Supplement: S2 Table — shows the adjusted prevalence of passive smoking among males and females by selected characteristics (gender, age, marriage, education and occupation) in 2001 and 2010. P1 is for 2001 vs. 2010; P2 is for the comparison of characteristics groups. (DOC) [file pone.0118500.s003.doc]

**S2 Table. Adjusted prevalence (95%CI) of passive smoking among males and females by selected characteristics (2001-2010)**

|  | Total | | | Male | | | Female | | |
| --- | --- | --- | --- | --- | --- | --- | --- | --- | --- |
| Characteristics | 2001 survey (n=2277) | 2010 survey (n=2102) | P1 | 2001 survey (n=943) | 2010 survey (n=848) | P1 | 2001 survey (n=1334) | 2010 survey (n=1254) | P1 |
|  |
|  | %(95%CI) | %(95%CI) |  | %(95%CI) | %(95%CI) |  | %(95%CI) | %(95%CI) |  |
| Total | 30.5(28.6-32.4) | 30.0(28.0-31.9) | 0.714 | 19.6(17.1-22.2) | 20.3(17.5-23.0) | 0.760 | 38.1(35.5-40.7) | 36.5(33.9-39.2) | 0.417 |
| Age group |  |  |  |  |  |  |  |  |  |
| 60- | 33.3(30.8-35.7) | 32.2(29.4-35.0) | 0.574 | 23.3(19.9-26.7) | 18.4(14.7-22.0) | 0.058 | 40.5(37.2-43.9) | 41.6(37.8-45.5) | 0.671 |
| 70- | 25.5(22.3-28.8) | 28.9(25.6-32.1) | 0.153 | 13.3(9.3-17.2) | 23.8(18.9-28.7) | **0.001** | 33.9(29.3-38.5) | 32.2(27.9-36.5) | 0.604 |
| 80- | 27.6(20.8-34.4) | 23.8(18.6-28.9) | 0.364 | 13.2(4.3-22.0) | 18.3(11.0-25.5) | 0.379 | 35.5(26.4-44.6) | 27.9(20.6-35.1) | 0.188 |
| P2 | **0.001** | **0.020** |  | **0.001** | 0.173 |  | 0.064 | **<0.001** |  |
| Marital status |  |  |  |  |  |  |  |  |  |
| Married | 30.9(28.8-33.0) | 29.4(27.3-31.5) | 0.316 | 20.0(17.4-22.7) | 19.6(16.8-22.4) | 0.808 | 40.2(37.2-43.2) | 37.2(34.2-40.2) | 0.162 |
| Widowed or divorced | 28.2(23.7-32.7) | 33.0(27.8-38.3) | 0.169 | 14.9(6.5-23.3) | 29.5(17.1-41.9) | 0.059 | 31.2(26.1-36.3) | 33.8(28.0-39.6) | 0.528 |
| P2 | 0.303 | 0.190 |  | 0.328 | **0.004** |  | 0.089 | 0.304 |  |
| Occupation |  |  |  |  |  |  |  |  |  |
| White collar | 25.1(22.5-27.6) | 27.1(23.9-30.4) | 0.331 | 20.6(17.4-23.8) | 21.4(16.6-26.1) | 0.774 | 30.8(26.7-35.0) | 31.1(26.7-35.6) | 0.943 |
| Light physical labor | 37.5(34.6-40.5) | 27.6(24.5-30.8) | **<0.001** | 20.7(15.5-25.8) | 19.2(15.2-23.1) | 0.606 | 42.7(39.2-46.2) | 35.8(31.1-40.6) | **0.024** |
| Hard physical labor | 21.5(14.8-28.3) | 36.2(32.4-40.0) | **0.001** | 9.5(3.0-16.1) | 20.8(14.7-26.9) | **0.033** | 36.1(24.2-48.0) | 42.3(37.7-46.9) | 0.422 |
| P2 | **<0.001** | **<0.001** |  | 0.067 | 0.746 |  | **<0.001** | **0.003** |  |
| Education level (years) |  |  |  |  |  |  |  |  |  |
| 0-6 | 35.3(32.3-38.3) | 38.1(33.9-42.3) | 0.280 | 16.6(12.0-21.2) | 31.7(23.3-40.2) | **0.001** | 41.9(38.3-45.5) | 40.0(35.2-44.8) | 0.522 |
| 7-12 | 28.5(25.3-31.6) | 31.6(28.6-34.7) | 0.153 | 20.6(16.4-24.9) | 19.1(14.6-23.5) | 0.619 | 34.8(30.3-39.3) | 38.1(34.1-42.1) | 0.279 |
| 13~ | 24.4(20.6-28.1) | 21.9(18.8-24.9) | 0.297 | 21.0(16.6-25.3) | 17.9(14.3-21.5) | 0.282 | 30.6(23.8-37.4) | 28.1(22.7-33.4) | 0.539 |
| P2 | **<0.001** | **<0.001** |  | 0.376 | **0.003** |  | **0.005** | **0.004** |  |

*P1 is for 2001 vs. 2010;*

*P2 is for the comparison of characteristics groups.*
