# Supplementary material for: Changes in and Patterns of Smoking Exposure in an Elderly Urban Population in Beijing: 2001–2010
Source: PLoS One. 2015 Mar 18;10(3):e0118500. doi: 10.1371/journal.pone.0118500 (PMC4364981; doi:10.1371/journal.pone.0118500)
Supplement: S3 Table — shows the pattern of active smoke exposure (CPD, SI and years of active smoke) and passive smoke exposure (CPD (P), years of passive smoke, the places and sources of passive smoking) in the 2001 and 2010 surveys; P is for 2001 vs. 2010. Data are mean (SD) for continuous values or % (95%CI) for category value; CPD = cigarettes per day; SI = age of smoking initiation; CPD (P) = cigarettes per day of passive smoking. (DOC) [file pone.0118500.s004.doc]

**S3 Table. Adjusted smoking status in the participants of two surveys in 2001 and 2010**

|  | Male | | | Female | | | Total | | |
| --- | --- | --- | --- | --- | --- | --- | --- | --- | --- |
| Mean（SD） | 2001(n=943) | 2010(n=848) | P | 2001(n=1334) | 2010(n=1254) | P | 2001(n=2277) | 2010(n=2102) | P |
| CPD | 14.8(9.1) | 14.3(10.7) | 0.413 | 8.5(7.3) | 10.1(10.4) | 0.114 | 13.2(9.1) | 13.5(10.8) | 0.588 |
| SI | 22.2(8.7) | 24.0(7.6) | **<0.001** | 21.6(11.9) | 27.9(12.8) | **<0.001** | 22.0(9.6) | 24.7(8.9) | **<0.001** |
| Lasting time | 34.6(14.1) | 36.2(13.4) | 0.075 | 34.0(18.6) | 35.7(17.3) | 0.460 | 34.5(15.4) | 36.1(14.2) | 0.053 |
| CPD(P) | 10.2(7.9) | 9.8(7.8) | 0.597 | 12.4(8.3) | 11.1(10.7) | **0.040** | 11.8(8.2) | 10.7(10.0) | **0.037** |
| Lasting time(P) | 18.1(14.7) | 20.3(14.2) | 0.136 | 28.9(14.5) | 29.7(14.9) | 0.418 | 26.0(15.3) | 27.1(15.3) | 0.187 |
| %(95%CI) |  |  |  |  |  |  |  |  |  |
| Active smoking status |  |  | 0.119 |  |  | **<0.001** |  |  | **<0.001** |
| current | 24.3(21.5-27.0) | 23.6(20.7-26.5) | 0.740 | 8.9(7.4-10.5) | 3.9(2.8-5.0) | **<0.001** | 15.3(13.8-16.7) | 11.8(10.5-13.2) | **0.001** |
| former | 33.9(30.9-36.9) | 31.4(28.2-34.5) | 0.261 | 4.6(3.5-5.8) | 4.5(3.4-5.7) | 0.911 | 16.7(15.2-18.2) | 15.3(13.8-16.9) | 0.218 |
| Never | 41.9(38.7-45.0) | 45.1(41.7-48.4) | 0.182 | 86.4(84.6-88.3) | 91.6(90.1-93.1) | **<0.001** | 68.0(66.1-70.0) | 72.8(70.9-74.7) | **0.001** |
| Passive smoking | 19.6(17.1-22.2) | 20.3(17.5-23.0) | 0.760 | 38.1(35.5-40.7) | 36.5(33.9-39.2) | 0.417 | 30.5(28.6-32.4) | 30.0(28.0-31.9) | 0.714 |
| Places |  |  |  |  |  |  |  |  |  |
| Family | 13.2(11.0-15.4) | 15.3(12.8-17.7) | 0.199 | 35.9(33.3-38.4) | 34.1(31.5-36.8) | 0.367 | 26.5(24.7-28.3) | 26.5(24.7-28.4) | 0.989 |
| Work | 5.2(3.8-6.6) | 3.8(2.5-5.1) | 0.144 | 0.7(0.3-1.2) | 1.5(0.9-2.2) | 0.064 | 2.6(1.9-3.2) | 2.5(1.8-3.1) | 0.875 |
| Public | 1.3(0.6-2.0) | 1.1(0.4-1.9) | 0.85 | 1.5(0.9-2.2) | 0.9(0.4-1.4) | 0.148 | 1.4(0.9-1.9) | 1.0(0.6-1.4) | 0.166 |
| Source |  |  |  |  |  |  |  |  |  |
| Couple | 5.8(4.3-7.3) | 2.1(1.1-3.0) | **<0.001** | 30.5(28.0-33.0) | 18.2(16.0-20.3) | **<0.001** | 20.3(18.7-22.0) | 11.7(10.3-13.0) | **<0.001** |
| Off-springs | 7.3(5.6-8.9) | 13.2(10.9-15.5) | **<0.001** | 5.3(4.1-6.5) | 15.2(13.2-17.2) | **<0.001** | 6.1(5.1-7.1) | 14.4(12.9-15.9) | **<0.001** |
| colleagues | 5.3(3.9-6.7) | 4.2(2.9-5.6) | 0.287 | 0.6(0.2-1.1) | 2.0(1.2-2.8) | **0.003** | 2.6(1.9-3.2) | 2.9(2.2-3.6) | 0.473 |
| others | 1.3(0.6-2.0) | 0.8(0.2-1.4) | 0.352 | 1.7(1.0-2.3) | 1.2(0.6-1.8) | 0.335 | 1.5(1.0-2.0) | 1.0(0.6-1.4) | 0.142 |

*P is for 2001 vs. 2010;*

*Data are mean (SD) for continuous values or % (95%CI) for category value;*

*CPD=* *cigarettes per day;*

*SI=* *age of smoking initiation;*

*CPD (P) = cigarettes per day of passive smoking*
